# Association between D-dimer to lymphocyte ratio and in hospital all-cause mortality in elderly patients with sepsis: a cohort of 1123 patients

**DOI:** 10.3389/fcimb.2024.1507992

**Published:** 2025-01-14

**Authors:** Xinguang Long, Zhenkui Hu, Chao Song, Jinhui Zhang

**Affiliations:** ^1^ Department of Cardiology, Yangzhong People’s Hospital, YangZhong, Jiangsu, China; ^2^ Department of Emergency Medicine, The Affiliated Hospital, Jiangsu University, Zhenjiang, Jiangsu, China; ^3^ Department of Critical Care Medicine, The Affiliated Hospital, Jiangsu University, Zhenjiang, Jiangsu, China

**Keywords:** sepsis, d-dimer, lymphocyte, D-dimer to lymphocyte ratio, all-cause mortality

## Abstract

**Background:**

The D-dimer to lymphocyte ratio (DLR), a novel inflammatory biomarker, had been shown to be related to adverse outcomes in patients with various diseases. However, there was limited research on the relationship between the DLR and adverse outcomes in patients with infectious diseases, particularly those with sepsis. Therefore, this study aimed to explore the association between the DLR and in hospital all-cause mortality in elderly patients with sepsis.

**Methods:**

A total of 1123 patients admitted in intensive care unit (ICU) were included in this study. The patients were categorized into quartiles (Q1-Q4) based on their DLR values. The primary outcomes included hospital mortality and ICU mortality. Kaplan-Meier analysis was conducted to compare all-cause mortality among the four DLR groups. The association between DLR and all-cause mortality in patients with sepsis was further elucidated using the receiver operating characteristic (ROC) curve and Cox proportional hazards regression analysis.

**Results:**

The study included participants with a median age of 75 (65-84) years, with 707 (63.0%) being male. The rates of hospital mortality and ICU mortality were 33.7% and 31.9%, respectively. Kaplan-Meier analysis highlighted a significantly increased risk of all-cause mortality among patients with elevated DLR values (log-rank p < 0.001). ROC curve analyses revealed that DLR had a stronger ability to predict hospital mortality and ICU mortality in patients with sepsis than D-dimer or Lym. Multivariable Cox proportional hazards analyses revealed DLR as an independent predictor of hospital death [per 1 SD increase in DLR: HR (95% CI): 1.098 (1.020-1.181); p = 0.013] and ICU death [per 1 SD increase in DLR: HR (95% CI): 1.095 (1.017-1.180); p = 0.017] during the hospital stay.

**Conclusions:**

A higher DLR value was associated with hospital and ICU all-cause death in elderly patients with sepsis. This finding demonstrated that the DLR could be a convenient and useful prognostic marker for sepsis prognosis.

## Introduction

Sepsis, a life-threatening condition characterized by organ dysfunction resulting from a dysregulated host response to infection, presented a significant challenge in intensive care unit (ICU) due to its high morbidity and mortality rates (Singer et al., 2016). Globally, approximately 30 million individuals were diagnosed with sepsis annually, with over 5 million succumbing to the condition (Fleischmann et al., 2016). Sepsis mortality rates could reach up to 60% in some developing nations, while developed countries reported rates ranging from 20% to 30% (Fleischmann et al., 2016; Hotchkiss et al., 2016; Shankar-Hari et al., 2018). Survivors of sepsis often faced long-term complications, including heightened long-term mortality, susceptibility to reinfection, and higher rates of hospital readmission (Prescott et al., 2016). The rapid progression and high mortality of sepsis contributed to its substantial socioeconomic burden, posing clinical challenges in its management (Prescott et al., 2016). To enhance patient outcomes in sepsis, it was imperative for researchers to identify accurate predictive factors that can inform therapy decisions. Previous studies had identified several prognostic factors such as soluble intercellular adhesion molecule 1 (sICAM-1), High-Mobility Group Box 1 (HMGB1), Prokineticin 2, Plasminogen Activator Inhibitor-1 (PAI-1), and claudin-5 (CLDN-5) (Hoshino et al., 2017; Brück et al., 2021; Yu et al., 2022; He et al., 2024). However, most of them were too expensive to carry out well in clinical practice. Thus, identify appliable prognostic factors for the sepsis remained challenging.

Although numerous studies had delved into the pathogenesis of sepsis, the precise mechanism remained unclear (Font et al., 2020; Liu et al., 2022). After the onset of sepsis, the function of T cells, B cells, and other immune cells (such as monocytes and macrophages) gradually declined due to persistent inflammatory stimulation and the release of cytokines (Kappelmayer et al., 2024). In this immunosuppressive state, the ability of immune cells to clear infections diminishes, allowing pathogens to persist and stimulate the coagulation system (Jacobi, 2022). Additionally, microthrombus formation caused by coagulopathy could hinder the arrival of immune cells at the site of infection, further weakening the immune response (Samuels et al., 2018). There was a complex interaction between immunosuppression and coagulation mechanisms in sepsis, with both contributing to disease progression and deterioration (Ge et al., 2024). Previous research had demonstrated that patients with sepsis experienced various degrees of coagulation dysfunction (Amaral et al., 2004; Semeraro et al., 2010). D-dimer, a coagulation factor, significantly increased during sepsis onset and had been utilized as a marker for sepsis severity (Han et al., 2021). Most studies suggested that coagulation activation, as reflected by elevated D-dimer levels, significantly contributed to the outcome of sepsis (Rodelo et al., 2012). Lymphocyte, as immune-inflammatory cell, played a crucial role in reflecting immune ability and inflammatory status (Martínez-Lostao et al., 2015). Lymphopenia, a common feature of sepsis-induced immunosuppression, hindered microbial clearance and predisposed individuals to serious infections, which were a leading cause of sepsis-related mortality (Drewry et al., 2014). Several studies had reported a decrease in peripheral blood lymphocyte count in sepsis patients, with lymphopenia identified as a risk factor for poor prognosis in sepsis (Cilloniz et al., 2021).

Notably, the D-dimer to lymphocyte ratio (DLR), a newly inflammatory composite marker, had exhibited predictive efficacy in evaluating mortality among patients diagnosed with Coronavirus Disease 2019 (COVID-19), ST-segment elevation myocardial infarction (STEMI), acute aortic dissection, and assessing the likelihood of liver metastasis in colorectal cancer patients (Peng et al., 2022; Lu et al., 2023; Xu et al., 2023; Cao et al., 2024). Despite these correlations, it remained uncertain whether DLR could predict adverse outcomes in patients with sepsis. Therefore, our study aimed to investigate the prognostic value of DLR regarding the risk of in hospital all-cause mortality in elderly patients with sepsis.

## Methods

### Study population

This was a retrospective cohort study conducted on elderly patients with sepsis hospitalized at the ICU of the Affiliated Hospital of Jiangsu University between January 2015 and November 2023. Patients who met the criteria for sepsis at the time of ICU admission were eligible for enrollment. Sepsis was defined according to the diagnostic criteria of Sepsis 3.0 (Font et al., 2020) (defined as lifethreatening organ dysfunction caused by a dysregulated host response to infection, with organ dysfunction identified as an acute change in total SOFA score ≥ 2 points). Only data from the patient’s first admission was used if they had multiple admission records. Exclusion criteria were applied, excluding patients under 18 years old, patients with an ICU length of stay less than 24h, patients with chronic kidney disease (CKD), patients with hepatic cirrhosis, patients with other comorbidities that might cause lymphopenia, such as malignant tumors, malnutrition, HIV infection, autoimmune diseases, immunosuppressive drugs, cytotoxic agents. Additionally, patients with insufficient data for analysis were also excluded. Following these criteria, a total of 1,123 elderly patients with sepsis hospitalized at the ICU were included in this retrospective cohort study. All patients were initially admitted to the ICU, and of these, 780 were subsequently transferred from the ICU to the general ward. The study protocol was approved by the ethics committee of the Affiliated Hospital of Jiangsu University (No. KY2023K1007), and the requirement for informed consent was waived because of its retrospective design.

### Data collection

The clinical variables utilized in this research were acquired from the electronic medical records. These variables could be divided into seven main groups: (1) demographics, such as age, gender, body mass index (BMI), and smoking status. (2) comorbidities, including hypertension, diabetes, coronary artery disease, chronic obstructive pulmonary disease (COPD), and cerebral infarction. (3) infection pathogens, including Gram-positive bacterial infection, Gram-negative bacterial infection, fungal infection, and viral infection; another group of patients was categorized as “others” because a pathogen was not identified. Gram-positive bacterial infections, Gram-negative bacterial infections, and fungal infections were detected using matrix assisted laser desorption ionization-time of flight mass spectrometry (MALDI-TOF MS), while viral infections were detected using quantitative fluorescence RT-PCR technology. (4) infection locations, including multisite infection, lower respiratory infection, gastrointestinal infection, intra-abdominal infection, genitourinary tract infection, bacteremia, and skin and soft tissue infection. (5) laboratory indicators, including white blood cell (WBC), neutrophil (Neu), lymphocyte (Lym), monocyte (Mon), hemoglobin (Hb), platelet (PLT), C-reactive protein (CRP), total bilirubin (Tbil), alanine transaminase (ALT), aspartate aminotransferase (AST), albumin, glucose, creatinine, blood urea nitrogen (BUN), uric acid, D-dimer, potassium, and lactate. (6) severity of illness scores, such as the Acute Physiology and Chronic Health Evaluation II (APACHE II) score and SOFA score. (7) treatments, including continuous renal replacement therapy (CRRT), vasoactive drugs, and invasive ventilation. Follow-up duration commenced on the date of admission and concluded on the date of discharge. All laboratory indicators and disease severity scores were derived from data collected within the initial 24 hours after the patient’s admission to the ICU. The DLR was calculated using the following formula: DLR = {D-dimer (mg/L)}/{lymphocyte count (10^^9^ \ cells/L)}. Subsequently, patients were divided into four groups based on their DLR quartile ranges: [Q1: (DLR ≤ 3.23, ≤ 25th percentile); Q2 (3.23 < DLR ≤ 8.18, 25th-50th percentile); Q3 (8.18 < DLR ≤ 20.12, 50th-75th percentile); Q4 (DLR > 20.12, > 75th percentile)].

### Clinical outcomes

The primary outcome of this study was in hospital all-cause mortality. Of these, hospital death included ICU death and general ward inpatient death. Secondary outcomes comprised the occurrence of acute kidney injury (AKI) and the ICU and hospital length of stay (LOS). AKI was defined by the 2012 Kidney Disease: Improving Global Outcomes Clinical Practice Guidelines (KDIGO) (Kellum et al., 2013).

### Statistical analysis

Statistical analysis was conducted using SPSS version 26.0, MedCalc 20, GraphPad Prism 10.0, and R software version 4.1.3. Continuous variables were presented as mean ± standard deviation (SD) or median (interquartile range (IQR)) and analyzed using the Student’s t-test or the Mann-Whitney U test. Number (percentage) and chi-square tests were employed to describe and compare categorical variables. The correlations between DLR and the severity of illness scores were assessed using Spearman’s analysis. Kaplan-Meier survival analysis was used to estimate all-cause mortality among groups based on different levels of the DLR, and their differences were assessed through log-rank tests. Additionally, the predictive value of the DLR on mortality was assessed by the area under the receiver operating characteristic curve (AUROC). The association between the DLR and primary outcome was assessed with univariable and multivariable Cox proportional hazard models. For each outcome and exposure (SHR as either a continuous variable or a categorical variable), three models were implemented. Model 1 was an unadjusted analysis. model 2 was adjusted for age, gender, BMI, smoking, hypertension, diabetes, WBC, Neu, PLT, CRP, Alb; model 3 was adjusted for age, gender, BMI, smoking, hypertension, diabetes, WBC, Neu, PLT, CRP, Alb, creatinine, BUN, uric acid, lactate, APACHE II score, and SOFA score. Furthermore, restricted cubic spline (RCS) regression with three knots (10th, 50th, and 90th percentiles) was applied to analyze the non-linearity association between DLR and hospital and ICU all-cause death. Further stratified analyses were performed based on age (≤65 or >65), gender (male or female), smoking (yes or no), hypertension (yes or no), diabetes (yes or no), lactate level (≤2.0 or >2.0), and AKI (yes or no) to examine the consistency of the prognostic value of the DLR for primary outcomes. The interaction between DLR and stratified variables was further tested. To assess the association between the DLR and secondary outcomes, multivariate binary logistic or linear regression analysis was employed. A p-value of less than 0.05 was considered statistically significant.

## Results

### Study population

**Figure 1:**
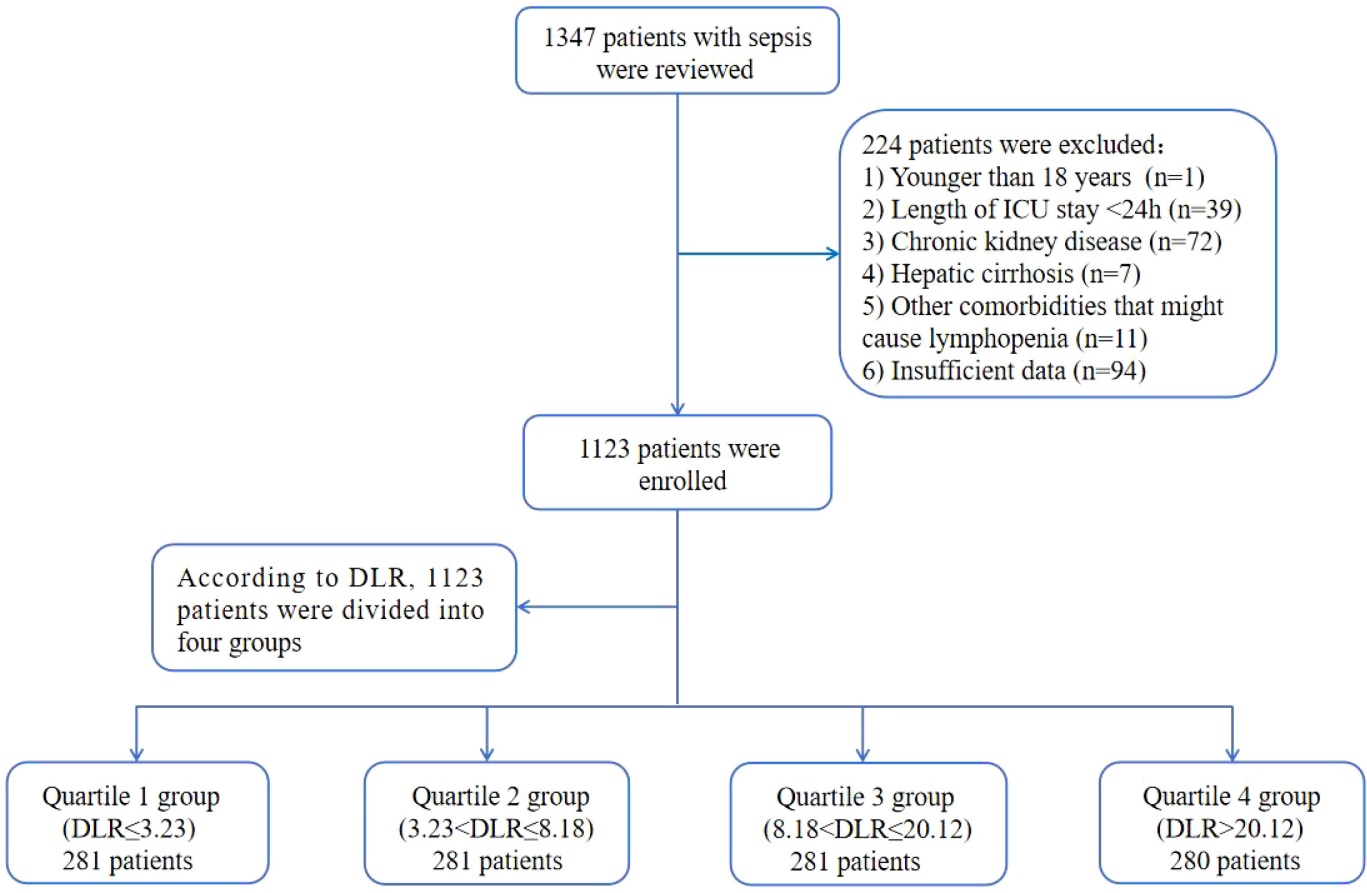
Flow of included patients through the trial. DLR, D-dimer to lymphocyte ratio; ICU, Intensive Care Unit.

The patient screening process was illustrated in **Figure 1**. A total of 1123 elderly patients with sepsis were included in the final analyses. The cohort had a median age of 75 (IQR: 65-84) years, with 707 (63.0%) being male. Among the participants, 579 (51.3%) had hypertension, 309 (27.5%) had diabetes, 116 (10.3%) had coronary artery disease, 87 (7.7%) had COPD, and 161 (14.3%) had cerebral infarction. Infection pathogens were detected in 48% of the patients, with Gram-negative bacteria (29.8) being the most prevalent, followed by Gram-positive bacteria (12.1%), fungi (6.9%), and viruses (5.3%). The median DLR for all participants was 8.18 (IQR: 3.23-20.12). The rates of hospital mortality and ICU mortality were 33.7% and 31.9%, respectively. A detailed summary of baseline and clinical characteristics was presented in **Table 1**.

**Table 1:** Characteristics and outcomes of participants categorized by DLR.

Variables	Overall	Q1 group (DLR ≤ 3.23)	Q2 group (3.23<DLR ≤ 8.18)	Q3 group (8.18<DLR ≤ 20.12)	Q4 group (DLR>20.12)	p-value
N	1123	281	281	281	280	
Age, years	75 (65-84)	74 (60-83)	75 (64-84)	77 (68-85)	77 (66-85)	0.020
Male, n (%)	707 (63.0)	183 (65.1)	171 (60.9)	194 (69.0)	159 (56.8)	0.018
BMI, kg/m^2^	22.49 (20.08-25.21)	23.12 (20.31-25.98)	22.65 (20.02-25.62)	22.22 (19.85-24.63)	22.04 (20.22-24.81)	0.014
Smoking, n (%)	229 (20.4)	54 (19.2)	60 (21.4)	65 (23.1)	50 (17.9)	0.433
Comorbidities, n (%)						
Hypertension	579 (51.3)	163 (58.0)	153 (54.4)	132 (47.0)	131 (46.8)	0.015
Diabetes	309 (27.5)	88 (31.3)	89 (31.7)	63 (22.4)	69 (24.6)	0.026
Coronary artery disease	116 (10.3)	27 (9.6)	28 (10.0)	32 (11.4)	29 (10.4)	0.914
COPD	87 (7.7)	26 (9.3)	21 (7.5)	26 (9.3)	14 (5.0)	0.190
Cerebral infarction	161 (14.3)	53 (18.9)	39 (13.9)	40 (14.2)	29 (10.4)	0.039
Infection pathogens, n (%)						
Gram-positive bacteria	136 (12.1)	35 (12.5)	33 (11.7)	40 (14.2)	28 (10.0)	0.488
Gram-negative bacteria	335 (29.8)	60 (21.4)	78 (27.8)	87 (31.0)	110 (39.3)	<0.001
Fungus	77 (6.9)	18 (6.4)	10 (3.6)	21 (7.5)	28 (10.0)	0.025
Virus	60 (5.3)	22 (7.8)	11 (3.9)	13 (4.6)	14 (5.0)	0.178
Others	515 (45.9)	146 (52.0)	149 (53.0)	120 (42.7)	100 (35.7)	<0.001
Infection sites, n (%)						
Multisite Infection	120 (10.7)	35 (12.5)	23 (8.2)	34 (12.1)	28 (10.0)	0.322
Lower respiratory infection	436 (38.8)	111 (39.5)	99 (35.2)	106 (37.7)	120 (42.9)	0.303
Gastrointestinal infection	11 (1.0)	3 (1.1)	2 (0.7)	4 (1.4)	1 (0.4)	0.571
Intra-abdominal infection	392 (34.9)	93 (33.1)	105 (37.4)	89 (31.7)	105 (37.5)	0.351
Genitourinary tract infection	69 (6.1)	19 (6.8)	22 (7.8)	19 (6.8)	9 (3.2)	0.115
Bacteremia	10 (0.9)	4 (1.4)	1 (0.4)	3 (1.1)	2 (0.7)	0.570
Skin and soft tissue infection	85 (7.6)	18 (6.4)	22 (7.8)	24 (8.5)	21 (7.5)	0.813
Laboratory tests						
WBC *10^9/^L	11.4 (7.4-17.1)	11.5 (8.2-17.6)	11.4 (7.9-16.6)	12.0 (6.9-16.9)	10.6 (6.5-17.3)	0.175
Neu *10^9/^L	10.1 (6.3-15.5)	9.8 (6.5-15.3)	9.9 (6.6-15.0)	10.7 (6.3-16.1)	9.8 (6.0-16.3)	0.823
Lym *10^9/^L	0.6 (0.3-0.9)	1.0 (0.6-1.4)	0.6 (0.4-1.0)	0.5 (0.3-0.6)	0.3 (0.2-0.4)	<0.001
Mon *10^9/^L	0.4 (0.2-0.7)	0.6 (0.3-0.8)	0.5 (0.3-0.7)	0.4 (0.2-0.6)	0.3 (0.2-0.5)	<0.001
Hb, g/dL	115 (97-130)	119 (104-134)	116 (95-133)	112 (99-129)	110 (93-125)	<0.001
PLT *10^9/^L	149 (95-214)	194 (133-262)	161 (114-225)	135 (89-193)	109 (68-166)	<0.001
CRP, mg/L	104.2 (42.0-163.2)	76.8 (18.4-120.8)	99.9 (36.4-166.0)	115.0 (54.3-178.4)	124.5 (64.1-187.6)	<0.001
Tbil, μmol/L	17.4 (10.9-28.2)	13.3 (8.2-22.1)	16.8 (10.5-27.1)	18.6 (12.1-30.3)	21.0 (13.4-35.8)	<0.001
ALT, U/L	32.0 (21.0-56.0)	30.0 (20.0-48.6)	30.0 (20.0-50.1)	33.0 (22.0-61.1)	37.0 (23.0-85.8)	<0.001
AST, U/L	38.1 (23.9-73.0)	30.3 (20.0-58.6)	35.0 (24.0-62.5)	44.0 (25.9-89.5)	52.5 (29.3-164.0)	<0.001
Alb, g/L	28.2 (24.2-33.2)	31.6 (27.0-36.0)	27.9 (23.8-32.6)	27.1 (24.2-31.7)	27.4 (22.9-31.2)	<0.001
Glucose, mmol/L	8.2 (6.6-11.8)	7.9 (6.4-10.9)	8.4 (6.6-12.8)	8.5 (6.7-12.0)	8.2 (6.5-11.7)	0.150
Creatinine, μmol/L	92.6 (63.7-153.1)	78.4 (55.6-117.5)	86.3 (58.8-145.8)	99.4 (63.5-154.4)	126.0 (78.2-201.1)	<0.001
BUN, mmol/L	8.89 (6.04-13.95)	7.16 (5.27-10.44)	8.32 (5.62-12.66)	9.64 (6.42-15.11)	11.83 (7.20-18.60)	<0.001
Uric acid, μmol/L	286.9 (192.3-411.7)	274.4 (189.0-371.0)	287.8 (190.0-412.1)	264.7 (179.2-381.8)	327.6 (231.0-477.9)	<0.001
D-dimer, mg/L	4.2 (2.1-8.4)	1.5 (0.9-2.4)	3.3 (2.2-4.9)	5.8 (3.8-8.6)	11.8 (8.0-21.5)	<0.001
Potassium, mmol/L	3.7 (3.3-4.2)	3.6 (3.3-4.0)	3.8 (3.3-4.2)	3.7 (3.3-4.2)	3.7 (3.3-4.3)	0.252
Lactate, mmol/L	2.1 (1.4-3.6)	1.8 (1.2-2.4)	2.1 (1.4-3.1)	2.2 (1.4-4.1)	2.8 (1.9-5.0)	<0.001
DLR	8.18 (3.23-20.12)	1.74 (1.00-2.42)	5.25 (4.08-6.53)	12.57 (9.90-15.38)	37.01 (26.91-67.66)	<0.001
Severity scoring						
APACHE II score	25 (19-30)	24 (19-30)	25 (18-29)	25 (19-29)	27 (22-33)	<0.001
SOFA score	12 (10-14)	11 (9-13)	12 (9-14)	12 (10-14)	13 (11-15)	<0.001
Treatments						
CRRT, n (%)	78 (6.9)	8 (2.8)	16 (5.7)	20 (7.1)	34 (12.1)	<0.001
Vasoactive drug, n (%)	748 (66.6)	133 (47.3)	167 (59.4)	216 (76.9)	232 (82.9)	<0.001
Invasive ventilation, n (%)	752 (67.0)	174 (61.9)	181 (64.4)	200 (71.2)	197 (70.4)	0.051
Endpoints						
30-day mortality, n (%)	316 (28.1)	40 (14.2)	65 (23.1)	88 (31.3)	123 (43.9)	<0.001
60-day mortality, n (%)	375 (33.4)	50 (17.8)	76 (27.0)	112 (39.9)	137 (48.9)	<0.001
AKI, n (%)	512 (45.6)	80 (28.5)	117 (41.6)	133 (47.3)	182 (65.0)	<0.001
ICU length of stay, days	6 (3-12)	6 (3-11)	5 (3-11)	6 (3-13)	6 (3-12)	0.120
Hospital length of stay, days	16 (11-25)	16 (11-24)	17 (11-26)	18 (11-28)	16 (10-24)	0.162
ICU mortality, n (%)	358 (31.9)	50 (17.8)	70 (24.9)	106 (37.7)	132 (47.4)	<0.001
Hospital mortality, n (%)	379 (33.7)	53 (18.9)	74 (26.3)	113 (40.2)	139 (49.6)	<0.001

DLR, D-dimer to lymphocyte ratio; BMI, body mass index; COPD, chronic obstructive pulmonary disease; WBC, white blood cell count; Neu, neutrophil; Lym, lymphocyte; Mon, monocyte; Hb, hemoglobin; PLT, platelet; CRP, C-reactive protein; Tbil, total bilirubin; ALT, alanine transaminase; AST, aspartate aminotransferase; Alb, albumin; BUN, blood urea nitroge; APACHE II, Acute Physiology and Chronic Health Evaluation II; SOFA, Sequential Organ Failure Assessment; CRRT, continuous renal replacement therapy; AKI, Acute kidney injury; ICU, Intensive Care Unit.

### Baseline characteristics

Baseline characteristics categorized according to quartiles of the DLR were outlined in **Table 1**. The median DLR values for each quartile were 1.74 (IQR: 1.00-2.42), 5.25 (IQR: 4.08-6.53), 12.57 (IQR: 9.00-15.38), and 37.01 (IQR: 26.91-67.66), respectively. Patients in the highest DLR quartile tended to be elder and female, with a higher prevalence of Gram-negative bacteria and fungus. They also exhibited elevated levels of CRP, Tbil, ALT, AST, creatinine, BUN, uric acid, D-dimer, and lactate, along with lower levels of BMI, Lym, Mon, Hb, PLT, and Alb. Additionally, they presented with higher severity scores, and a higher proportion required CRRT, and vasoactive drugs compared to the lower DLR groups. Spearman’s correlation analysis revealed positive correlations between DLR and APACHE II score (0.112, p < 0.001) as well as between DLR and SOFA score (0.132, p < 0.001) (**Supplementary Figure 1**). With increasing DLR, there was a gradual rise in the 30-day mortality (14.2% vs. 23.1% vs. 31.3% vs. 43.9%, p < 0.001), 60-day mortality (17.8% vs. 27.0% vs. 39.9% vs. 48.9%, p < 0.001), ICU mortality (17.8% vs. 24.9% vs. 37.7% vs. 47.4%, p < 0.001), and hospital mortality (18.9% vs. 26.3% vs. 40.2% vs. 49.6%, p < 0.001). Given the better association of the Q4 group with all-cause mortality, we furthered compared the difference between Q4 and Q1-3. This analysis revealed consistent results across different grouping approaches (**Supplementary Table 1**).

### Association between the all-cause mortality and DLR

**Figure 2:**
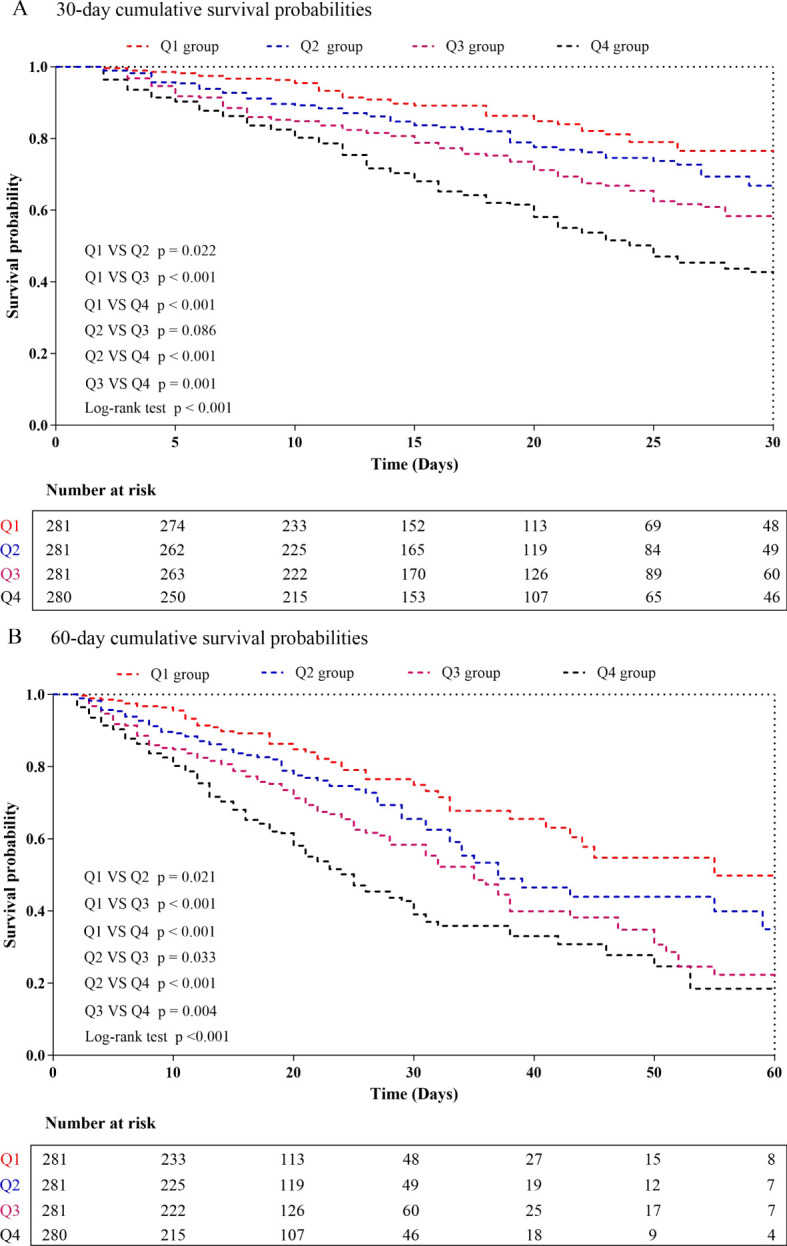
Kaplan-Meier curves showing cumulative probability of all-cause mortality according to groups at 30 days **(A)**, and 60 days **(B)**. DLR quartiles: Q1 group (DLR ≤ 3.23); Q2 group (3.23<DLR ≤ 8.18); Q3 group (8.18<DLR ≤ 20.12); Q4 group (DLR>20.12).

**Figure 3:**
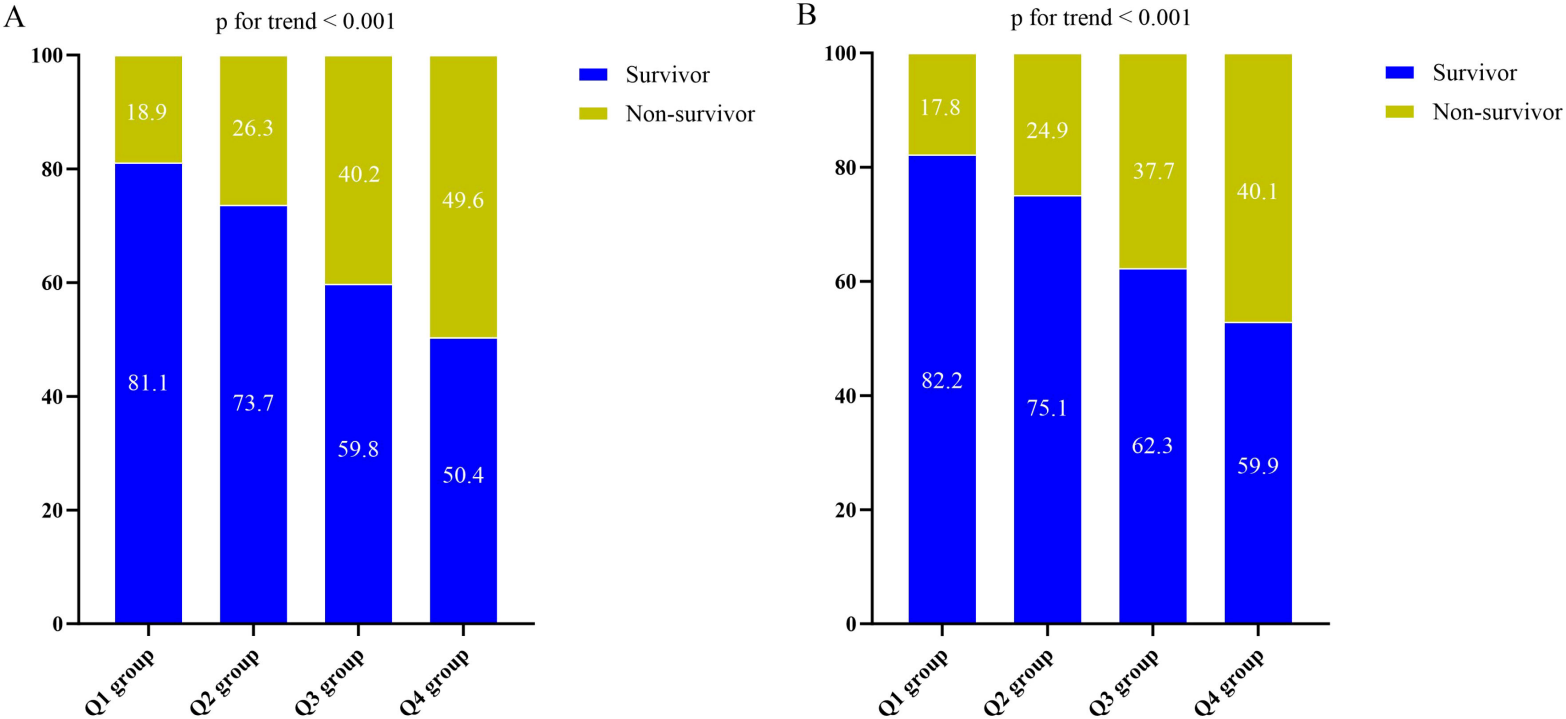
**(A)** The prevalence of hospital mortality ratio among different quartiles of DLR. **(B)** The prevalence of ICU mortality ratio among different quartiles of DLR. DLR quartiles: Q1 group (DLR ≤ 3.23); Q2 group (3.23<DLR ≤ 8.18); Q3 group (8.18<DLR ≤ 20.12); Q4 group (DLR>20.12). DLR, D-dimer to lymphocyte ratio; ICU, Intensive Care Unit.

**Figure 4:**
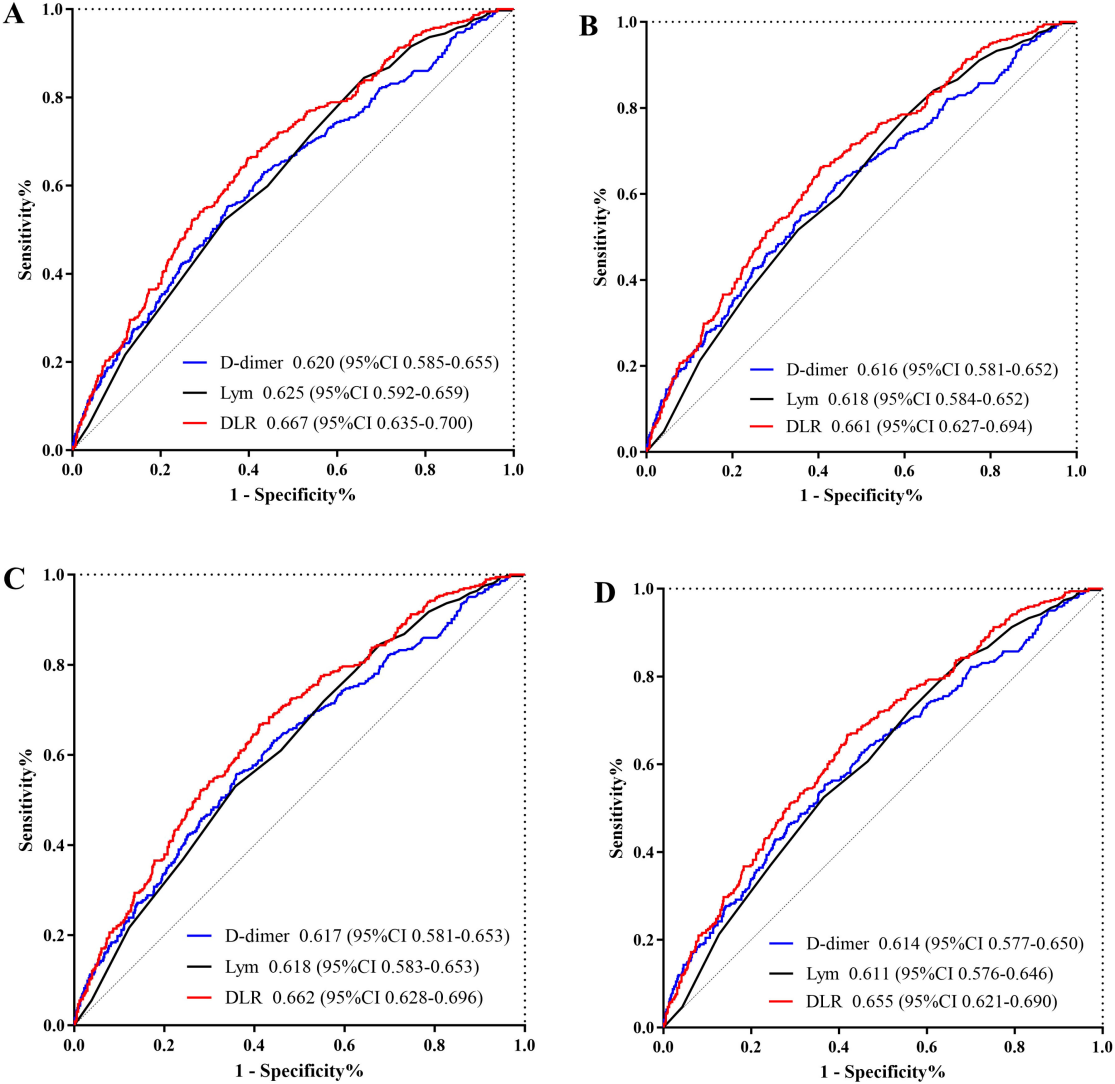
**(A)** ROC curve analysis of the DLR to predict hospital mortality and comparison of the AUC between the DLR, Lym, and D-dimer. p value is =0.009 (DLR v.s. Lym), and p value is <0.001 (DLR v.s. D-dimer); **(B)** ROC curve analysis of the DLR to predict ICU mortality and comparison of the AUC between the DLR, Lym, and D-dimer. p value is =0.010 (DLR v.s. Lym), and p value is <0.001 (DLR v.s. D-dimer); **(C)** ROC curve analysis of the DLR to predict hospital mortality in elderly patients (aged ≥65 years) and comparison of the AUC between the DLR, Lym, and D-dimer. p value is =0.009 (DLR v.s. Lym), and p value is <0.001 (DLR v.s. D-dimer); **(D)** ROC curve analysis of the DLR to predict ICU mortality in elderly patients (aged ≥65 years) and comparison of the AUC between the DLR, Lym, and D-dimer. p value is =0.010 (DLR v.s. Lym), and p value is <0.001 (DLR v.s. D-dimer). DLR, D-dimer to lymphocyte ratio; Lym, lymphocyte; ICU, Intensive Care Unit; ROC, receiver operating characteristic curve.

The Kaplan-Meier survival analysis curves for assessing the incidence of 30-day mortality among groups based on the quartile groupings of the DLR were shown in **Figure 2A**. There was a statistically significant difference in 30-day mortality rate in the groups (log-rank test, p <0.001). Similar results were observed for the prediction of 60-day mortality (log-rank test, p <0.001) (**Figure 2B**). Additionally, **Figures 3A, B** indicated an increasing trend in all-cause mortality with higher DLR. ROC curve analyses were performed to assess the predictive value of DLR, Lym, and D-dimer in all-cause mortality (**Figures 4A, B**). The results indicated that the AUC of the DLR for predicting hospital mortality and ICU mortality was 0.667 (95%CI: 0.635-0.700) and 0.661 (95%CI: 0.627-0.694), respectively. Furthermore, the ROC curve analysis demonstrated that the DLR outperformed Lym (p for comparison = 0.009 for hospital death; p for comparison = 0.010 for ICU death) or D-dimer (p for comparison < 0.001 for hospital death; p for comparison < 0.001 for ICU death). Similar results were observed in predicting hospital death and ICU death among elderly patients (aged ≥ 65 years) (**Figures 4C, D**).

**Figure 5:**
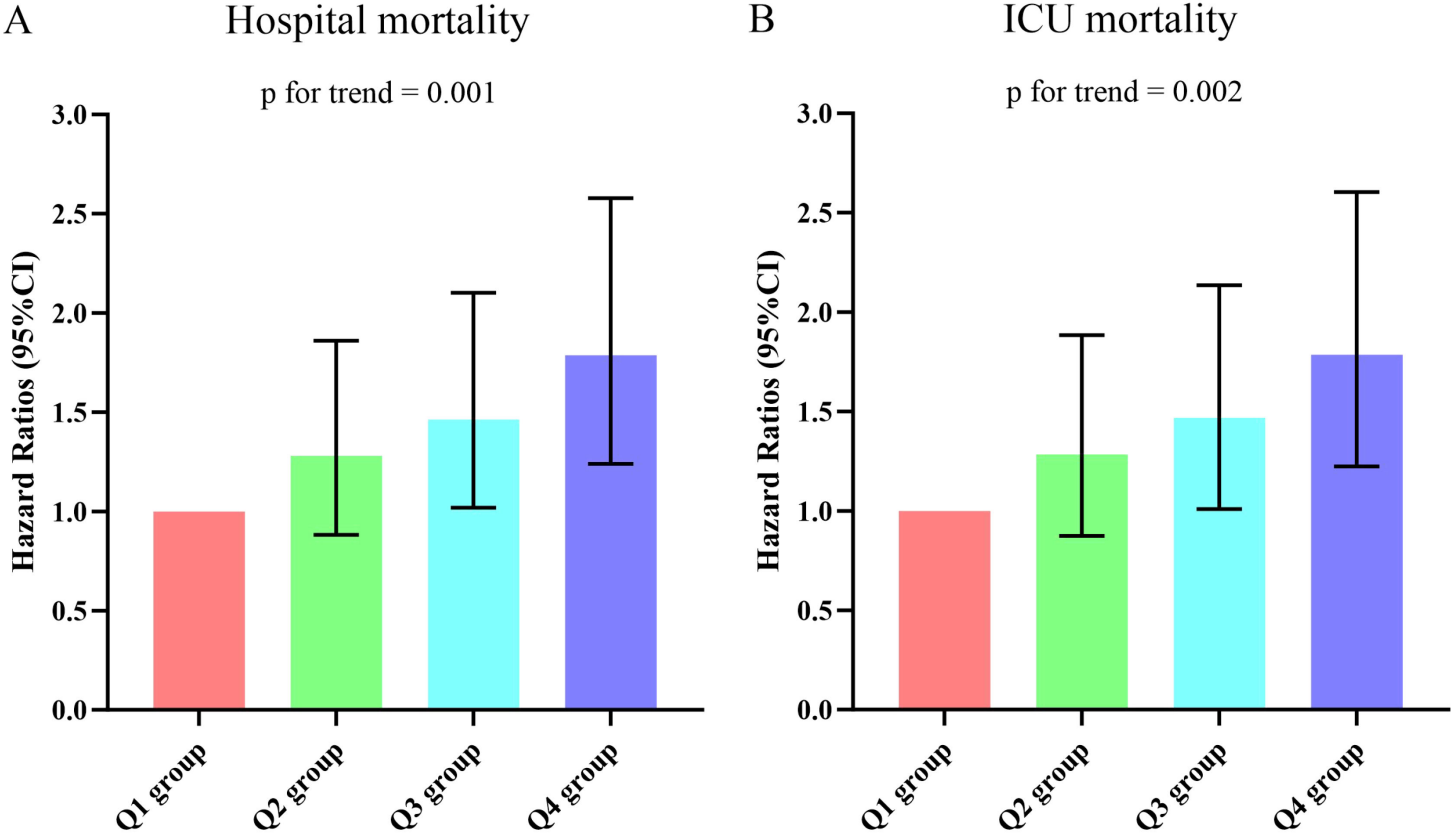
**(A, B)** Hazard ratios (95% CIs) for hospital/ICU mortality according to DLR quartiles after adjusting for age, gender, BMI, Smoking, hypertension, diabetes, WBC, Neu, PLT, CRP, Alb, creatinine, BUN, uric acid, lactate, APACHE II score,and SOFA score. Error bars indicate 95% CIs. The first quartile is the reference. DLR quartiles: Q1 group (DLR ≤ 3.23); Q2 group (3.23<DLR ≤ 8.18); Q3 group (8.18<DLR ≤ 20.12); Q4 group (DLR>20.12). DLR, D-dimer to lymphocyte ratio; BMI, body mass index; WBC, white blood cell; Neu, neutrophil; PLT, platelet; CRP, C-reactive protein; Alb, albumin; BUN, blood urea nitroge; APACHE II, Acute Physiology and Chronic Health Evaluation II; SOFA, Sequential Organ Failure Assessment; ICU, Intensive Care Unit.

**Supplementary Table 2** presented the outcomes of univariate COX regression analysis assessing the risk of all-cause death in elderly patients with sepsis and variables with a significance level of p < 0.05 in the univariate analysis, and factors influencing prognosis suggested by clinicians were considered as independent variables for univariate COX regression analysis. The influential factors included age, male, BMI, smoking, hypertension, diabetes, WBC, Neu, PLT, CRP, Alb, creatinine, BUN, uric acid, lactate, APACHE II score, and SOFA score. Multivariable COX regression analysis was employed to examine the association between DLR and all-cause death, as outlined in **Table 2**. In three models, whether increased by 1 unit or 1 SD, the DLR was significantly correlated with hospital death and ICU death. The risk of hospital death for DLR Q2, Q3, and Q4 was higher than DLR Q1, indicating an increasing trend with DLR [Q1 vs. Q2: HR (95%CI): 1.281 (0.882-1.860); Q3: HR (95%CI): 1.463 (1.018-2.102); Q4: HR (95%CI): 1.787 (1.239-2.578); p for trend=0.001] (**Figure 5A**). Similar results were observed in the Cox proportional risk analysis of DLR and ICU death [Q1 vs. Q2: HR (95%CI): 1.284 (0.875-1.885); Q3: HR (95%CI): 1.468 (1.010-2.135); Q4: HR (95%CI): 1.786 (1.224-2.604); p for trend=0.002] (**Figure 5B**).

**Table 2:** Cox proportional hazard ratios (HR) for all-cause mortality.

Variables	Model 1			Model 2			Model 3		
	HR (95% CI)	p-value	P for trend	HR (95% CI)	p-value	P for trend	HR (95% CI)	p-value	P for trend
Hospital mortality									
Continuous variable per unit	1.005 (1.003-1.006)	<0.001		1.004 (1.002-1.005)	<0.001		1.002 (1.000-1.004)	0.013	
Continuous variable per SD	1.211 (1.138-1.289)	<0.001		1.158 (1.083-1.283)	<0.001		1.098 (1.020-1.181)	0.013	
Quartile^a^			<0.001			<0.001			0.001
Q1 group	Ref			Ref			Ref		
Q2 group	1.428 (1.003-2.034)	0.048		1.331 (0.925-1.917)	0.124		1.281 (0.882-1.860)	0.193	
Q3 group	1.997 (1.441-2.769)	<0.001		1.605 (1.134-2.272)	0.008		1.463 (1.018-2.102)	0.039	
Q4 group	2.942 (2.142-4.041)	<0.001		2.349 (1.658-3.327)	<0.001		1.787 (1.239-2.578)	0.002	
ICU mortality									
Continuous variable per unit	1.005 (1.003-1.006)	<0.001		1.004 (1.002-1.005)	<0.001		1.002 (1.000-1.004)	0.017	
Continuous variable per SD	1.211 (1.137-1.290)	<0.001		1.158 (1.082-1.240)	<0.001		1.095 (1.017-1.180)	0.017	
Quartile^a^			<0.001			<0.001			0.002
Q1 group	Ref			Ref			Ref		
Q2 group	1.425 (0.990-2.050)	0.056		1.331 (0.915-1.935)	0.135		1.284 (0.875-1.885)	0.202	
Q3 group	1.989 (1.420-2.785)	<0.001		1.608 (1.124-2.299)	0.009		1.468 (1.010-2.135)	0.044	
Q4 group	2.941 (2.122-4.075)	<0.001		2.359 (1.649-3.374)	<0.001		1.786 (1.224-2.604)	0.003	

Model 1: unadjusted.

Model 2: adjusted for age, gender, BMI, smoking, hypertension, diabetes, WBC, Neu, PLT, CRP, Alb.

Model 3: adjusted for age, gender, BMI, Smoking, hypertension, diabetes, WBC, Neu, PLT, CRP, Alb, creatinine, BUN, uric acid, lactate, APACHE II score,and SOFA score.

^a^DLR: Q1 group (DLR ≤ 3.23); Q2 group (3.23<DLR ≤ 8.18); Q3 group (8.18<DLR ≤ 20.12); Q4 group (DLR>20.12).

DLR, D-dimer to lymphocyte ratio; BMI, body mass index; WBC, white blood cell; Neu, neutrophil; PLT, platelet; CRP, C-reactive protein; Alb, albumin; BUN, blood urea nitroge; APACHE II, Acute Physiology and Chronic Health Evaluation II; SOFA, Sequential Organ Failure Assessment; ICU, Intensive Care Unit.

After adjusting for possible confounding factors, RCS curve was performed (**Supplementary Figure 2**). We confirmed that the correlation between DLR and hospital death and ICU death was nonlinear after adjusting age, male, BMI, smoking, hypertension, diabetes, WBC, Neu, PLT, CRP, Alb, creatinine, BUN, uric acid, lactate, APACHE II score, and SOFA score. We calculated the infection point as 22.5 using two-piecewise linear regression and a recursive algorithm (**Supplementary Table 3**). DLR was positively correlated with hospital death and ICU death to the left of the infection point. There was no increased mortality on the right of the infection point as DLR increased.

### Subgroup analysis

To further analyzed the relationship between DLR and all-cause mortality in elderly patients with sepsis, we categorized the population based on age (≤65 or >65), gender (male or female), smoking (yes or no), hypertension (yes or no), diabetes (yes or no), lactate (≤2.0 or >2.0), and AKI (yes or no). Multivariable Cox regression analysis revealed no significant interactions between age, gender, smoking, hypertension, diabetes, lactate, and AKI for all-cause mortality (all p for interaction > 0.05) (**Tables 3**, **4**). Although no interaction was found between DLR and lactate, statistical significance was observed only among patients with lactate level > 2.0 mmol/L.

**Table 3:** Subgroup analysis regarding the influence of different DLR in the hospital mortality.

Subgroups	No. hospital mortality/No. patients	HR (95% CI)	p-value	P for interaction
Age				0.697
>65	315/840	1.005 (1.003-1.006)	<0.001	
≤65	64/283	1.005 (1.001-1.010)	0.029	
Gender				0.606
Male	251/707	1.005 (1.003-1.007)	<0.001	
Female	128/416	1.004 (1.001-1.007)	0.002	
Smoking				0.081
Yes	87/229	1.010 (1.005-1.015)	<0.001	
No	291/893	1.004 (1.003-1.006)	<0.001	
Hypertension				0.940
Yes	211/579	1.005 (1.003-1.007)	<0.001	
No	168/544	1.005 (1.003-1.007)	<0.001	
Diabetes				0.621
Yes	111/309	1.004 (1.002-1.007)	0.001	
No	268/814	1.005 (1.003-1.007)	<0.001	
Lactate				0.866
>2.0	277/616	1.004 (1.002-1.005)	<0.001	
≤2.0	102/507	1.005 (1.000-1.010)	0.058	
AKI				0.368
Yes	223/512	1.003 (1.002-1.005)	<0.001	
No	156/611	1.006 (1.002-1.009)	0.003	

DLR, D-dimer to lymphocyte ratio; AKI, Acute kidney injury.

**Table 4:** Subgroup analysis regarding the influence of different DLR in the ICU mortality.

Subgroups	No. ICU mortality/No. patients	HR (95% CI)	p-value	P for interaction
Age				0.793
>65	297/840	1.005 (1.003-1.006)	<0.001	
≤65	61/283	1.005 (1.000-1.010)	0.039	
Gender				0.606
Male	238/707	1.005 (1.003-1.007)	<0.001	
Female	120/416	1.004 (1.002-1.007)	0.002	
Smoking				0.076
Yes	84/229	1.010 (1.005-1.015)	<0.001	
No	273/893	1.004 (1.003-1.006)	<0.001	
Hypertension				0.962
Yes	199/579	1.005 (1.003-1.007)	<0.001	
No	159/544	1.005 (1.002-1.007)	<0.001	
Diabetes				0.598
Yes	104/309	1.004 (1.002-1.007)	0.001	
No	254/814	1.005 (1.003-1.007)	<0.001	
Lactate				0.899
>2.0	266/616	1.004 (1.002-1.005)	<0.001	
≤2.0	92/507	1.004 (0.998-1.010)	0.168	
AKI				0.455
Yes	212/512	1.004 (1.002-1.005)	<0.001	
No	146/611	1.005 (1.001-1.009)	0.007	

DLR, D-dimer to lymphocyte ratio; AKI, Acute kidney injury; ICU, Intensive Care Unit.

### Association between DLR and secondary outcomes

Univariate analysis revealed that the highest DLR group was significantly associated with an increased risk of AKI occurrence when compared to the other groups (28.5% vs. 41.6% vs. 47.3% vs. 65.0%, p < 0.001). However, the analysis of ICU and hospital length of stay (LOS) among the four groups did not reveal significant differences (**Table 1**). After adjusting for confounding factors (age, male, BMI, smoking, hypertension, diabetes, WBC, Neu, PLT, CRP, Alb, BUN, lactate, APACHE II score, and SOFA score), the results indicated that an increase of either 1 unit or 1 SD in DLR was significantly and positively correlated with AKI occurrence (**Supplementary Table 4**).

## Discussion

This was the first investigation to examine the connection between DLR and sepsis prognosis in our knowledge. The findings suggested that elevated DLR was significantly associated with higher hospital mortality and ICU mortality even after adjustment for confounding variables. Moreover, the ROC curve analyses revealed that DLR had the best predictive value with a higher area under the curve than D-dimer or Lym for predict hospital mortality and ICU mortality. These results indicated that the DLR was an independent risk factor of poor prognosis in elderly patients with sepsis.

Sepsis is a disease that occurs when the body’s response to an infection becomes imbalanced, leading to organ dysfunction (Huang et al., 2019). Numerous studies had demonstrated strong associations between sepsis and various factors, including tissue damage, abnormal coagulation function, immune dysfunction, systemic inflammation, and genetic polymorphisms (Huang et al., 2019). Among these dysregulations, coagulopathy played a crucial role in the pathogenesis of sepsis-related dysregulated host response to infections (Semeraro et al., 2012; Mayer et al., 2015). Recent evidence suggested that acute disseminated intravascular coagulation (DIC) occured in approximately 25-50% of sepsis patients, significantly increasing the risk of mortality (Zeerleder et al., 2005; Voves et al., 2006; Gando et al., 2019; Patel et al., 2019). One of the most sensitive measures of coagulation, fibrin fragment D-dimer, was formed when plasmin cleaved insoluble fibrin, and its elevated levels predicted a higher risk of thrombosis (Chapin and Hajjar, 2015). Inflammatory cytokines released during sepsis enhanced the degradation of cross-linked fibrin polymers, leading to increased production of D-dimer (Fiusa et al., 2015). Elevated levels of D-dimer and fibrinogen degradation producted rapidly occur after DIC initiation, which can complicate sepsis (Trimaille et al., 2020). Several studies had demonstrated a significant association between D-dimer levels and poor outcomes in sepsis patients. For example, Rodelo et al. found that low D-dimer levels were associated with reduced survival in sepsis patients (Rodelo et al., 2012). Tang et al. observed a higher 28-day mortality rate in sepsis patients with elevated D-dimer levels during hospitalization (Tang et al., 2023). Schupp et al. reported that high D-dimer (> 30 mg/L) were associated with the highest risk of 30-day all-cause mortality in sepsis patients (Schupp et al., 2023). Moreover, Meini et al. demonstrated that D-dimer can help stratify the risk of in-hospital mortality and complications in patients with invasive infections caused by the Gram-negative bacteria Neisseria meningitidis (Meini et al., 2021). Another study involving 268 sepsis patients suggested that an emergency department admission D-dimer level > 500 µg/ml independently associated with an increased short-term mortality rate (Innocenti et al., 2019).

Lymphocyte, essential components of the human immune system, played a crucial role in sepsis (Drewry et al., 2014). During pathogen infection, antigen-presenting cells recognized microbial antigens and presented them to T cells. CD4^+^ T cells then secreted cytokines that aided phagocytic cells in eliminating intracellular bacteria (Stearns-Kurosawa et al., 2011). Lymphocyte count declined significantly due to apoptosis in patients with sepsis (Le Tulzo et al., 2002). When sepsis occurs, pro-inflammatory factors and high mobility group box-1 protein (HMGB1) passively released from dead cells cause the up-regulation of programmed death-ligand 1 (PD-L1) through Toll-like Receptor 2 (TLR2) on neutrophils. The binding of PD-L1 and PD-1 on lymphocytes leads to increased apoptosis of lymphocytes and immune dysfunction, eventually resulting in the occurrence of sepsis immunosuppression (Liu et al., 2024). This lymphopenia was a common characteristic of sepsis-induced immunosuppression, hindering microbial clearance and increasing susceptibility to severe infections (Drewry et al., 2014). The severity and duration of lymphopenia were associated with worse clinical outcomes, including higher mortality rates (Wang et al., 2022). A meta-analysis of eight studies revealed that sepsis patients who died had significantly lower absolute lymphocyte counts compared to those who survived (Yang et al., 2024). These findings suggested that the absolute lymphocyte count could potentially serve as an indicator for predicting the prognosis of sepsis patients. In a prospective study involving 92 ICU-admitted sepsis patients, a decrease in the absolute lymphocyte count at baseline was linked to increased mortality rates (Chung et al., 2015). Another study utilizing data from the MIMIC-IV database, which included 1027 patients, reported that a decrease in the absolute lymphocyte count at baseline was associated with a higher incidence of 90-day mortality (Liu et al., 2023). Cilloniz et al. found that lymphopenia independently correlated with an increased risk of ICU admission, as well as higher in-hospital and 30-day mortality in sepsis patients (Cilloniz et al., 2021).

Numerous studies had highlighted the role of DLR in predicting outcomes in various diseases. For instance, studies had shown that a high admission DLR could serve as a robust predictor for increased in-hospital mortality among patients with acute aortic dissection (Xu et al., 2023). Similarly, a retrospective cohort study found that DLR could aid physicians in assessing the risk of liver metastasis in patients with colorectal cancer, predicting patient prognosis, and guiding treatment decisions more effectively (Lu et al., 2023). Furthermore, recent research demonstrated that DLR was a valuable predictor for the occurrence of major adverse cardiac events (MACEs) in patients with STEMI during hospitalization and long-term follow-up after percutaneous coronary intervention (PCI) (Cao et al., 2024). Peng et al. also suggested that DLR had a greater AUC compared to D-dimer or lymphocytes individually for predicting COVID-19-related mortality, indicating that combining D-dimer and lymphocytes might offer superior insights into the condition of COVID-19 patients (Peng et al., 2022). However, no studies had explored the relationship between DLR and clinical prognosis in elderly patients with sepsis. In our study, we discovered that DLR had a better ability to predict all-cause mortality in patients with sepsis than D-dimer or lymphocyte. Additionally, heightened DLR in sepsis patients were found to be correlated with an elevated risk of all-cause mortality. These findings suggested that early elevation of DLR might be useful in identifying elderly patients with sepsis at high risk of poor outcomes.

Furthermore, our study delved into risk stratification among various subgroups. Subgroup analysis indicated that the predictive value of DLR for in hospital all-cause mortality remained consistent across male and female patients. However, we did not observe a significant association between DLR and in hospital all-cause mortality among patients with hypertension or diabetes included in the study. This discrepancy might be attributed to reverse causality, wherein patients diagnosed with these comorbidities were more likely to have received appropriate treatment or adopted healthier lifestyle habits. Moreover, our study revealed that the association between DLR and all-cause mortality appeared to be more pronounced in patients with lactate levels exceeding 2.0 mmol/L, suggesting that hyperlactatemia may influence the predictive performance of DLR for all-cause mortality. Thus, maintaining optimal lactate levels could mitigate adverse clinical outcomes in sepsis patients. The APACHE II score or SOFA score were widely utilized indicators for assessing the severity of sepsis patients (Tekin et al., 2024). A high APACHE II score or SOFA score indicated severe infection that required aggressive anti-infection treatment (Liu et al., 2016; Liu et al., 2022). Our study indicated a positive correlation between DLR and the APACHE II score or SOFA score. In relation to secondary outcomes, an elevated DLR at the time of ICU admission emerged as a valuable inflammatory marker for evaluating the risk of AKI occurrence in elderly patients with sepsis. Consequently, it was crucial to provide timely intervention for patients with a high DLR value to prevent further deterioration.

This study had several limitations. Firstly, selection and confounding biases were challenging to avoid despite our best efforts to account for possible confounders and conduct subgroup analysis, which was an inherent drawback of retrospective investigations. Secondly, we only recorded the hospital all-cause mortality without conducting follow-up after discharge; therefore, the association between the DLR and long-term adverse events remained unknown in such populations. Thirdly, our study only examined DLR within 24 hours of ICU admission in patients with sepsis and failed to evaluate the dynamic effect of DLR. Fourth, sepsis can present with a wide variety of clinical features, influenced by patient demographics, underlying health conditions, and infection types. This variability can affect the immune response and overall clinical trajectory, which may impact the prognostic significance of DLR. Moreover, due to the complex interplay of multiple factors contributing to sepsis outcomes, certain unmeasured confounders, such as the timing of clinical intervention, severity of organ dysfunction, and treatment protocols, were not accounted for in the analysis and may impact the relationship between DLR and outcomes. Furthermore, the study focused on elderly patients with sepsis from China, and the association may not be fully generalizable to the general population or disease population. To overcome these limitations, more comprehensive data from broader studies with larger sample sizes and extended follow-up will be necessary to validate our findings and improve our understanding of the association between DLR and prognosis in elderly patients with sepsis.

## Conclusion

The findings from our study suggested that DLR served as a valuable indicator for predicting the risk of in hospital all-cause mortality and AKI occurrence in elderly patients with sepsis. Hence, incorporating DLR measurements into clinical practice could prove beneficial for assessing risk and predicting prognosis within this cohort. It’s imperative for future research endeavors to investigate whether interventions aimed at modulating DLR could enhance clinical outcomes for these patients.

## Data Availability

The raw data supporting the conclusions of this article will be made available by the authors, without undue reservation.

## References

[B1] Amaral A. Opal S. M. Vincent J. L. (2004). Coagulation in sepsis. Intensive Care Med. 30, 1032–1040. doi: 10.1007/s00134-004-2291-8 15148567

[B2] Brück E. Svensson-Raskh A. Larsson J. W. Caravaca A. S. Gallina A. L. Eberhardson M.. (2021). Plasma HMGB1 levels and physical performance in ICU survivors. Acta anaesthesiologica Scandinavica 65, 921–927. doi: 10.1111/aas.13815 33725363

[B3] Cao S. Liu Y. Ye J. Wang Y. Wang Z. Li C.. (2024). The value of D-dimer to lymphocyte ratio in predicting clinical outcomes after percutaneous coronary intervention in ST-segment elevation myocardial infarction patients: A retrospective study. Int. Immunopharmacol 128, 111556. doi: 10.1016/j.intimp.2024.111556 38241843

[B4] Chapin J. C. Hajjar K. A. (2015). Fibrinolysis and the control of blood coagulation. Blood Rev. 29, 17–24. doi: 10.1016/j.blre.2014.09.003 25294122 PMC4314363

[B5] Chung K. P. Chang H. T. Lo S. C. Chang L. Y. Lin S. Y. Cheng A.. (2015). Severe lymphopenia is associated with elevated plasma interleukin-15 levels and increased mortality during severe sepsis. Shock 43, 569–575. doi: 10.1097/SHK.0000000000000347 25692255

[B6] Cilloniz C. Peroni H. J. Gabarrús A. García-Vidal C. Pericàs J. M. Bermejo-Martin J.. (2021). Lymphopenia is associated with poor outcomes of patients with community-acquired pneumonia and sepsis. Open Forum Infect. Dis. 8, ofab169. doi: 10.1093/ofid/ofab169 34189165 PMC8231373

[B7] Drewry A. M. Samra N. Skrupky L. P. Fuller B. M. Compton S. M. Hotchkiss R. S. (2014). Persistent lymphopenia after diagnosis of sepsis predicts mortality. Shock 42, 383–391. doi: 10.1097/SHK.0000000000000234 25051284 PMC4362626

[B8] Fiusa M. M. Carvalho-Filho M. A. Annichino-Bizzacchi J. M. De Paula E. V. (2015). Causes and consequences of coagulation activation in sepsis: an evolutionary medicine perspective. BMC Med. 13, 105. doi: 10.1186/s12916-015-0327-2 25943883 PMC4422540

[B9] Fleischmann C. Scherag A. Adhikari N. K. Hartog C. S. Tsaganos T. Schlattmann P.. (2016). Assessment of global incidence and mortality of hospital-treated sepsis. Current estimates and limitations. Am. J. Respir. Crit. Care Med. 193, 259–272. doi: 10.1164/rccm.201504-0781OC 26414292

[B10] Font M. D. Thyagarajan B. Khanna A. K. (2020). Sepsis and Septic Shock - Basics of diagnosis, pathophysiology and clinical decision making. Med. Clin. North Am. 104, 573–585. doi: 10.1016/j.mcna.2020.02.011 32505253

[B11] Gando S. Shiraishi A. Yamakawa K. Ogura H. Saitoh D. Fujishima S.. (2019). Role of disseminated intravascular coagulation in severe sepsis. Thromb. Res. 178, 182–188. doi: 10.1016/j.thromres.2019.04.025 31054468

[B12] Ge J. Deng Q. Zhou R. Hu Y. Zhang X. Zheng Z. (2024). Identification of key biomarkers and therapeutic targets in sepsis through coagulation-related gene expression and immune pathway analysis. Front. Immunol. 15. doi: 10.3389/fimmu.2024.1470842 PMC11486639 39430765

[B13] Han Y. Q. Yan L. Zhang L. Ouyang P. H. Li P.. (2021). Performance of D-dimer for predicting sepsis mortality in the intensive care unit. Biochem. Med. (Zagreb) 31, 20709. doi: 10.11613/BM.2021.020709 PMC8183117 34140832

[B14] He R. R. Yue G. L. Dong M. L. Wang J. Q. Cheng C. (2024). Sepsis biomarkers: advancements and clinical applications-A narrative review. Int. J. Mol. Sci. 25, 9010. doi: 10.3390/ijms25169010 39201697 PMC11354379

[B15] Hoshino K. Kitamura T. Nakamura Y. Irie Y. Matsumoto N. Kawano Y.. (2017). Usefulness of plasminogen activator inhibitor-1 as a predictive marker of mortality in sepsis. J. Intensive Care 5, 42. doi: 10.1186/s40560-017-0238-8 28702197 PMC5504563

[B16] Hotchkiss R. S. Moldawer L. L. Opal S. M. Reinhart K. Turnbull I. R. Vincent J. L. (2016). Sepsis and septic shock. Nat. Rev. Dis. Primers 2, 16045. doi: 10.1038/nrdp.2016.45 28117397 PMC5538252

[B17] Huang M. Cai S. Su J. (2019). The pathogenesis of sepsis and potential therapeutic targets. Int. J. Mol. Sci. 20, 5376. doi: 10.3390/ijms20215376 31671729 PMC6862039

[B18] Innocenti F. Gori A. M. Giusti B. Tozzi C. Donnini C. Meo F.. (2019). Prognostic value of sepsis-induced coagulation abnormalities: an early assessment in the emergency department. Intern. Emerg. Med. 14, 459–466. doi: 10.1007/s11739-018-1990-z 30535649

[B19] Jacobi J. (2022). The pathophysiology of sepsis-2021 update: Part 1, immunology and coagulopathy leading to endothelial injury. Am. J. Health-System Pharmacy: AJHP: Off. J. Am. Soc. Health-System Pharmacists 79, 329–337. doi: 10.1093/ajhp/zxab380 PMC8500113 34605875

[B20] Kappelmayer J. Debreceni I. B. Fejes Z. Nagy B. Jr. (2024). Inflammation, sepsis, and the coagulation system. Hamostaseologie 44, 268–276. doi: 10.1055/a-2202-8544 38354835

[B21] Kellum J. A. Lameire N. KDIGO AKI Guideline Work Group (2013). Diagnosis, evaluation, and management of acute kidney injury: a KDIGO summary (Part 1). Crit. Care 17, 204. doi: 10.1186/cc11454 23394211 PMC4057151

[B22] Le Tulzo Y. Pangault C. Gacouin A. Guilloux V. Tribut O. Amiot L.. (2002). Early circulating lymphocyte apoptosis in human septic shock is associated with poor outcome. Shock 18, 487–494. doi: 10.1097/00024382-200212000-00001 12462554

[B23] Liu D. Huang S. Y. Sun J. H. Zhang H. C. Cai Q. L. Gao C.. (2022). Sepsis-induced immunosuppression: mechanisms, diagnosis and current treatment options. Mil Med. Res. 9, 56. doi: 10.1186/s40779-022-00422-y 36209190 PMC9547753

[B24] Liu X. Shen Y. Li Z. Fei A. Wang H. Ge Q.. (2016). Prognostic significance of APACHE II score and plasma suPAR in Chinese patients with sepsis: a prospective observational study. BMC Anesthesiology 16, 46. doi: 10.1186/s12871-016-0212-3 27473112 PMC4966698

[B25] Liu J. Song K. Lin B. Chen Z. Zuo Z. Fang Y.. (2024). HMGB1 promotes neutrophil PD-L1 expression through TLR2 and mediates T cell apoptosis leading to immunosuppression in sepsis. Int. Immunopharmacol. 133, 112130. doi: 10.1016/j.intimp.2024.112130 38648712

[B26] Liu C. Suo S. Luo L. Chen X. Ling C. Cao S. (2022). SOFA score in relation to sepsis: clinical implications in diagnosis, treatment, and prognostic assessment. Comput. Math. Methods Med. 2022, 7870434. doi: 10.1155/2022/7870434 35991153 PMC9385349

[B27] Liu W. Tao Q. Xiao J. Du Y. Pan T. Wang Y.. (2023). Low lymphocyte to high-density lipoprotein ratio predicts mortality in sepsis patients. Front. Immunol. 14. doi: 10.3389/fimmu.2023.1279291 PMC10601636 37901205

[B28] Lu S. Gong S. Wu F. Ma L. Xiang B. Li L.. (2023). D-dimer to lymphocyte ratio can serve as a potential predictive and prognostic value in colorectal cancer patients with liver metastases. BMC Surg. 23, 64. doi: 10.1186/s12893-023-01958-z 36966285 PMC10040125

[B29] Martínez-Lostao L. Anel A. Pardo J. (2015). How do cytotoxic lymphocytes kill cancer cells? Clin. Cancer Res. 21, 5047–5056. doi: 10.1158/1078-0432.CCR-15-0685 26567364

[B30] Mayer C. L. Parello C. S. Lee B. C. Itagaki K. Kurosawa S. Stearns-Kurosawa D. J. (2015). Pro-coagulant endothelial dysfunction results from EHEC shiga toxins and host damage-associated molecular patterns. Front. Immunol. 6. doi: 10.3389/fimmu.2015.00155 PMC4387861 25904918

[B31] Meini S. Sozio E. Bertolino G. Sbrana F. Ripoli A. Pallotto C.. (2021). D-dimer as biomarker for early prediction of clinical outcomes in patients with severe invasive infections due to streptococcus pneumoniae and neisseria meningitidis. Front. Med. (Lausanne) 8. doi: 10.3389/fmed.2021.627830 PMC8081958 33937280

[B32] Patel P. Walborn A. Rondina M. Fareed J. Hoppensteadt D. (2019). Markers of inflammation and infection in sepsis and disseminated intravascular coagulation. Clin. Appl. Thromb. Hemost 25, 1076029619843338. doi: 10.1177/1076029619843338 30991817 PMC6714897

[B33] Peng F. Yi Q. Zhang Q. Deng J. Li C. Xu M.. (2022). Performance of D-dimer to lymphocyte ratio in predicting the mortality of COVID-19 patients. Front. Cell Infect. Microbiol. 12. doi: 10.3389/fcimb.2022.1053039 PMC9797859 36590587

[B34] Prescott H. C. Osterholzer J. J. Langa K. M. Angus D. C. Iwashyna T. J. (2016). Late mortality after sepsis: propensity matched cohort study. BMJ 353, i2375. doi: 10.1136/bmj.i2375 27189000 PMC4869794

[B35] Rodelo J. R. de la Rosa G. Valencia M. L. Ospina S. Arango C. M. Gómez C. I.. (2012). D-dimer is a significant prognostic factor in patients with suspected infection and sepsis. Am. J. Emergency Med. 30, 1991–1999. doi: 10.1016/j.ajem.2012.04.033 22795996

[B36] Samuels J. M. Moore H. B. Moore E. E. (2018). Coagulopathy in severe sepsis: interconnectivity of coagulation and the immune system. Surg. Infections 1, 208–215. doi: 10.1089/sur.2017.260 PMC6425980 29346034

[B37] Schupp T. Weidner K. Rusnak J. Jawhar S. Forner J. Dulatahu F.. (2023). D-dimer levels and the disseminated intravascular coagulation score to predict severity and outcomes in sepsis or septic shock. Clin. Lab. 69. doi: 10.7754/Clin.Lab.2022.221015 37145079

[B38] Semeraro N. Ammollo C. T. Semeraro F. Colucci M. (2010). Sepsis-associated disseminated intravascular coagulation and thromboembolic disease. Mediterr J. Hematol. Infect. Dis. 2, e2010024. doi: 10.4084/MJHID.2010.024 21415977 PMC3033145

[B39] Semeraro N. Ammollo C. T. Semeraro F. Colucci M. (2012). Sepsis, thrombosis and organ dysfunction. Thromb. Res. 129, 290–295. doi: 10.1016/j.thromres.2011.10.013 22061311

[B40] Shankar-Hari M. Harrison D. A. Rowan K. M. Rubenfeld G. D. (2018). Estimating attributable fraction of mortality from sepsis to inform clinical trials. J. Crit. Care 45, 33–39. doi: 10.1016/j.jcrc.2018.01.018 29413720

[B41] Singer M. Deutschman C. S. Seymour C. W. Shankar-Hari M. Annane D. Bauer M.. (2016). The third international consensus definitions for sepsis and septic shock (Sepsis-3). JAMA 315, 801–810. doi: 10.1001/jama.2016.0287 26903338 PMC4968574

[B42] Stearns-Kurosawa D. J. Osuchowski M. F. Valentine C. Kurosawa S. Remick D. G. (2011). The pathogenesis of sepsis. Annu. Rev. Pathol. 6, 19–48. doi: 10.1146/annurev-pathol-011110-130327 20887193 PMC3684427

[B43] Tang J. Yuan H. Wu Y. L. Fu S. Pan X. Y. (2023). The predictive value of heparin-binding protein and D-dimer in patients with sepsis. Int. J. Gen. Med. 16, 2295–2303. doi: 10.2147/IJGM.S409328 37304904 PMC10257474

[B44] Tekin B. Kiliç J. Taşkin G. Solmaz İ. Tezel O. Başgöz B. B. (2024). The Comparison of scoring systems: SOFA, APACHE-II, LODS, MODS, and SAPS-II in critically ill elderly sepsis patients. J. Infect. Dev. Ctries 18, 122–130. doi: 10.3855/jidc.18526 38377099

[B45] Trimaille A. Thachil J. Marchandot B. Curtiaud A. Leonard-Lorant I. Carmona A.. (2020). D-dimers level as a possible marker of extravascular fibrinolysis in COVID-19 patients. J. Clin. Med. 10, 39. doi: 10.3390/jcm10010039 33374487 PMC7795726

[B46] Voves C. Wuillemin W. A. Zeerleder S. (2006). International Society on Thrombosis and Haemostasis score for overt disseminated intravascular coagulation predicts organ dysfunction and fatality in sepsis patients. Blood Coagul Fibrinolysis 17, 445–451. doi: 10.1097/01.mbc.0000240916.63521.2e 16905947

[B47] Wang G. Mivefroshan A. Yaghoobpoor S. Khanzadeh S. Siri G. Rahmani F.. (2022). Prognostic value of platelet to lymphocyte ratio in sepsis: A systematic review and meta-analysis. BioMed. Res. Int. 2022, 9056363. doi: 10.1155/2022/9056363 35707370 PMC9192240

[B48] Xu Y. Liang S. Liang Z. Huang C. Luo Y. Liang G.. (2023). Admission D-dimer to lymphocyte counts ratio as a novel biomarker for predicting the in-hospital mortality in patients with acute aortic dissection. BMC Cardiovasc. Disord. 23, 69. doi: 10.1186/s12872-023-03098-x 36740681 PMC9900915

[B49] Yang J. Zhu X. Feng J. (2024). The changes in the quantity of lymphocyte subpopulations during the process of sepsis. Int. J. Mol. Sci. 25, 1902. doi: 10.3390/ijms25031902 38339179 PMC10855580

[B50] Yu X. Chen J. Tang H. Tu Q. Li Y. Yuan X.. (2022). Identifying prokineticin2 as a novel immunomodulatory factor in diagnosis and treatment of sepsis. Crit. Care Med. 50, 674–684. doi: 10.1097/CCM.0000000000005335 34582411 PMC8923365

[B51] Zeerleder S. Hack C. E. Wuillemin W. A. (2005). Disseminated intravascular coagulation in sepsis. Chest 128, 2864–2875. doi: 10.1378/chest.128.4.2864 16236964

