# Supplementary material for: Association between D-dimer to lymphocyte ratio and in hospital all-cause mortality in elderly patients with sepsis: a cohort of 1123 patients
Source: Front Cell Infect Microbiol. 2025 Jan 14;14:1507992. doi: 10.3389/fcimb.2024.1507992 (PMC11772276; doi:10.3389/fcimb.2024.1507992)
Supplement: Supplementary file 1 [file DataSheet1.docx]

**Supplementary material**

Supplementary Table 1. Baseline characteristics of the Q1-Q3 and Q4 groups.

Supplementary Table 2. Cox proportional hazards regression of the factors influencing all-cause death of the study population.

Supplementary Table 3. Threshold effect analysis of DLR on all-cause mortality in ICU patients with sepsis using the two-piecewise model of linear regression.

Supplementary Table 4. Relationship between DLR and AKI occurrence.

Supplementary Figure 1. Association between DLR and APACHE II score and SOFA score using Spearman’s analysis.

Supplementary Figure 2. Restricted cubic spline regression analysis of DLR with in hospital all-cause mortality.

Table S1. Baseline characteristics of the Q1-Q3 and Q4 groups

| **Variables** | **Overall** | **Q1-Q3 group** | **Q4 group** | **P-value** |
| --- | --- | --- | --- | --- |
| N | 1123 |  | 280 |  |
| Age, years | 75 (65-84) | 75 (65-84) | 77 (66-85) | 0.117 |
| Male, n (%) | 707 (63.0) | 548 (65.0) | 159 (56.8) | 0.014 |
| BMI, kg/m^2^ | 22.49 (20.08-25.21) | 22.49 (20.03-25.39) | 22.04 (20.22-24.81) | 0.174 |
| Smoking, n (%) | 229 (20.4) | 179 (21.2) | 50 (17.9) | 0.234 |
| **Comorbidities, n (%)** | | | | |
| Hypertension | 579 (51.3) | 448 (53.1) | 131 (46.8) | 0.065 |
| Diabetes | 309 (27.5) | 240 (28.5) | 69 (24.6) | 0.214 |
| Coronary artery disease | 116 (10.3) | 87 (10.3) | 29 (10.4) | 0.991 |
| COPD | 87 (7.7) | 73 (8.7) | 14 (5.0) | 0.047 |
| Cerebral infarction | 161 (14.3) | 132 (15.7) | 29 (10.4) | 0.028 |
| **Infection pathogens, n (%)** | | | | |
| Gram-positive bacteria | 136 (12.1) | 108 (12.8) | 28 (10.0) | 0.212 |
| Gram-negative bacteria | 335 (29.8) | 225 (26.7) | 110 (39.3) | <0.001 |
| Fungus | 77 (6.9) | 49 (5.8) | 28 (10.0) | 0.016 |
| Virus | 60 (5.3) | 46 (5.5) | 14 (5.0) | 0.768 |
| **Laboratory tests** | | | | |
| WBC *10^9^ /L | 11.4 (7.4-17.1) | 11.5 (7.8-17.0) | 10.6 (6.5-17.3) | 0.078 |
| Neu *10^9^ /L | 10.1 (6.3-15.5) | 10.1 (6.5-15.4) | 9.8 (6.0-16.3) | 0.565 |
| Lym *10^9^ /L | 0.6 (0.3-0.9) | 0.6 (0.4-1.0) | 0.3 (0.2-0.4) | <0.001 |
| Mon *10^9^ /L | 0.4 (0.2-0.7) | 0.5 (0.2-0.7) | 0.3 (0.2-0.5) | <0.001 |
| Hb, g/dL | 115 (97-130) | 117 (99-132) | 110 (93-125) | <0.001 |
| PLT *10^9^ /L | 149 (95-214) | 164 (109-228) | 109 (68-166) | <0.001 |
| CRP, mg/L | 104.2 (42.0-163.2) | 96.6 (36.9-159.3) | 124.5 (64.1-187.6) | <0.001 |
| Tbil, μmol/L | 17.4 (10.9-28.2) | 16.7 (10.1-26.1) | 21.0 (13.4-35.8) | <0.001 |
| ALT, U/L | 32.0 (21.0-56.0) | 31.0 (21.0-51.0) | 37.0 (23.0-85.8) | <0.001 |
| AST, U/L | 38.1 (23.9-73.0) | 36.0 (23.0-66.0) | 52.5 (29.3-164.0) | <0.001 |
| Alb, g/L | 28.2 (24.2-33.2) | 29.0 (24.6-33.6) | 27.4 (22.9-31.2) | <0.001 |
| Glucose, mmol/L | 8.2 (6.6-11.8) | 8.2 (6.6-11.8) | 8.2 (6.5-11.7) | 0.936 |
| Creatinine, μmol/L | 92.6 (63.7-153.1) | 84.9 (59.4-135.3) | 126.0 (78.2-201.1) | <0.001 |
| BUN, mmol/L | 8.89 (6.04-13.95) | 8.34 (5.72-12.02) | 11.83 (7.20-18.60) | <0.001 |
| Uric acid, μmol/L | 286.9 (192.3-411.7) | 275.3 (187.0-397.4) | 327.6 (231.0-477.9) | <0.001 |
| D-dimer, mg/L | 4.2 (2.1-8.4) | 3.1 (1.7-5.4) | 11.8 (8.0-21.5) | <0.001 |
| Potassium, mmol/L | 3.7 (3.3-4.2) | 3.7 (3.3-4.1) | 3.7 (3.3-4.3) | 0.981 |
| Lactate, mmol/L | 2.1 (1.4-3.6) | 2.0 (1.3-3.1) | 2.8 (1.9-5.0) | <0.001 |
| DLR | 8.18 (3.23-20.12) | 5.25 (2.42-9.9) | 37.01 (26.91-67.66) | <0.001 |
| **Severity scoring** |  |  |  |  |
| APACHE II score | 25 (19-30) | 25 (19-30) | 27 (22-33) | <0.001 |
| SOFA score | 12 (10-14) | 12 (9-11) | 13 (11-15) | <0.001 |
| **Treatments** | | | | |
| CRRT, n (%) | 78 (6.9) | 44 (5.2) | 34 (12.1) | <0.001 |
| Vasoactive drug, n (%) | 748 (66.6) | 516 (61.2) | 232 (82.9) | <0.001 |
| Invasive ventilation, n (%) | 752 (67.0) | 555 (65.8) | 197 (70.4) | 0.163 |
| **Endpoints** | | | | |
| 30-day mortality, n (%) | 316 (28.1) | 193 (22.9) | 123 (43.9) | <0.001 |
| 60-day mortality, n (%) | 375 (33.4) | 238 (28.2) | 137 (48.9) | <0.001 |
| AKI, n (%) | 512 (45.6) | 330 (39.1) | 182 (65.0) | <0.001 |
| Length of ICU stay, days | 6 (3-12) | 6 (3-11) | 6 (3-12) | 0.335 |
| Length of hospital stay, days | 16 (11-25) | 17 (11-26) | 16 (10-24) | 0.052 |
| ICU mortality, n (%) | 358 (31.9) | 226 (26.8) | 132 (47.4) | <0.001 |
| Hospital mortality, n (%) | 379 (33.7) | 240 (28.5) | 139 (49.6) | <0.001 |

Abbreviations: DLR, D-dimer to lymphocyte ratio; BMI, body mass index; COPD, chronic obstructive pulmonary disease; WBC, white blood cell count; Neu, neutrophil; Lym, lymphocyte; Mon, monocyte; Hb, hemoglobin; PLT, platelet; CRP, C-reactive protein; Tbil, total bilirubin; ALT, alanine transaminase; AST, aspartate aminotransferase; Alb, albumin; BUN, blood urea nitroge; APACHE II, Acute Physiology and Chronic Health Evaluation II; SOFA, Sequential Organ Failure Assessment; CRRT, continuous renal replacement therapy; AKI, Acute kidney injury; ICU, Intensive Care Unit.

Table S2. Cox proportional hazards regression of the factors influencing all cause mortality of the study population.

| **Variables** | **HR** | **95% CI** | **P-value** |
| --- | --- | --- | --- |
| DLR | 1.005 | 1.003-1.006 | <0.001 |
| Age | 1.019 | 1.011-1.027 | <0.001 |
| Male | 0.953 | 0.770-1.180 | 0.660 |
| BMI | 0.976 | 0.953-1.000 | 0.048 |
| Smoking | 1.145 | 0.901-1.456 | 0.269 |
| Hypertension | 1.161 | 0.948-1.423 | 0.149 |
| Diabetes | 1.153 | 0.924-1.439 | 0.206 |
| WBC | 1.014 | 1.001-1.026 | 0.028 |
| Neu | 1.019 | 1.005-1.032 | 0.005 |
| CRP | 1.002 | 1.000-1.003 | 0.006 |
| Alb | 0.985 | 0.970-1.000 | 0.044 |
| Creatinine | 1.001 | 1.001-1.002 | <0.001 |
| BUN | 1.027 | 1.021-1.034 | <0.001 |
| Uric acid | 1.001 | 1.001-1.002 | <0.001 |
| Lactate | 1.100 | 1.073-1.128 | <0.001 |
| APACHE II score | 1.044 | 1.032-1.057 | <0.001 |
| SOFA score | 1.078 | 1.052-1.105 | <0.001 |

Abbreviations: DLR, D-dimer to lymphocyte ratio; BMI, body mass index; WBC, white blood cell; Neu, neutrophil; PLT, platelet; CRP, C-reactive protein; Alb, albumin; BUN, blood urea nitroge; APACHE II, Acute Physiology and Chronic Health Evaluation II; SOFA, Sequential Organ Failure Assessment; ICU, Intensive Care Unit.

Table S3. Threshold effect analysis of DLR on all-cause mortality in ICU patients with sepsis using the two-piecewise model of linear regression

| **Variables** | **Adjusted HR** | **95%CI** | **P-value** |
| --- | --- | --- | --- |
| **Hospital mortality** | | | |
| Fitting by the standard linear model | 1.002 | 1.000-1.004 | 0.013 |
| Fitting by the two-piecewise linear model |  |  |  |
| Infection point | 22.5 |  |  |
| DLR<=22.5 | 1.044 | 1.014-1.075 | 0.004 |
| DLR>22.5 | 1.002 | 1.000-1.005 | 0.056 |
| Log likelihood ratio |  |  | <0.001 |
| **ICU mortality** | | | |
| Fitting by the standard linear model | 1.002 | 1.000-1.004 | 0.017 |
| Fitting by the two-piecewise linear model |  |  |  |
| Infection point | 22.5 |  |  |
| DLR<=22.5 | 1.037 | 1.006-1.068 | 0.018 |
| DLR>22.5 | 1.002 | 1.000-1.005 | 0.066 |
| Log likelihood ratio |  |  | <0.001 |

adjusted for age, gender, BMI, Smoking, hypertension, diabetes, WBC, Neu, PLT, CRP, Alb, creatinine, BUN, uric acid, lactate, APACHE II score,and SOFA score.

Abbreviations: DLR, D-dimer to lymphocyte ratio; BMI, body mass index; WBC, white blood cell; Neu, neutrophil; PLT, platelet; CRP, C-reactive protein; Alb, albumin; BUN, blood urea nitroge; APACHE II, Acute Physiology and Chronic Health Evaluation II; SOFA, Sequential Organ Failure Assessment; ICU, Intensive Care Unit.

Table S4. Relationship between DLR and AKI occurrence

| **Variables** | **Model 1** | | | **Model 2** | | | **Model 3** | | |
| --- | --- | --- | --- | --- | --- | --- | --- | --- | --- |
|  | **HR (95% CI)** | **p-value** | **P for trend** | **HR (95% CI)** | **p-value** | **P for trend** | **HR (95% CI)** | **p-value** | **P for trend** |
| **AKI occurrence** | | | | | | | | | |
| Continuous variable per unit | 1.015 (1.010-1.020) | <0.001 |  | 1.011 (1.006-1.016) | <0.001 |  | 1.007 (1.002-1.012) | 0.009 |  |
| Continuous variable per SD | 1.826 (1.479-2.256) | <0.001 |  | 1.571 (1.281-1.927) | <0.001 |  | 1.335 (1.076-1.656) | 0.009 |  |
| Quartile^a^ |  |  | <0.001 |  |  | <0.001 |  |  | 0.015 |
| Q1 group | Ref |  |  | Ref |  |  | Ref |  |  |
| Q2 group | 1.792 (1.262-2.547) | 0.001 |  | 1.543 (1.059-2.249) | 0.024 |  | 1.413 (0.891-2.241) | 0.142 |  |
| Q3 group | 2.258 (1.592-3.202) | <0.001 |  | 1.738 (1.177-2.567) | 0.005 |  | 1.269 (0.777-2.074) | 0.342 |  |
| Q4 group | 4.666 (3.265-6.668) | <0.001 |  | 3.627 (2.428-5.416) | <0.001 |  | 2.018 (1.217-3.344) | 0.006 |  |

Model 1: unadjusted

Model 2: adjusted for age, gender, BMI, smoking, hypertension, diabetes, WBC, Neu, PLT, CRP, Alb.

Model 3: adjusted for age, gender, BMI, Smoking, hypertension, diabetes, WBC, Neu, PLT, CRP, Alb, BUN, lactate, APACHE II score,and SOFA score.

a DLR: Q1 group (DLR≤3.23); Q2 group (3.23<DLR≤8.18); Q3 group (8.18<DLR≤20.12); Q4 group (DLR>20.12).

Abbreviations: DLR, D-dimer to lymphocyte ratio; BMI, body mass index; WBC, white blood cell; Neu, neutrophil; PLT, platelet; CRP, C-reactive protein; Alb, albumin; BUN, blood urea nitroge; APACHE II, Acute Physiology and Chronic Health Evaluation II; SOFA, Sequential Organ Failure Assessment; ICU, Intensive Care Unit.


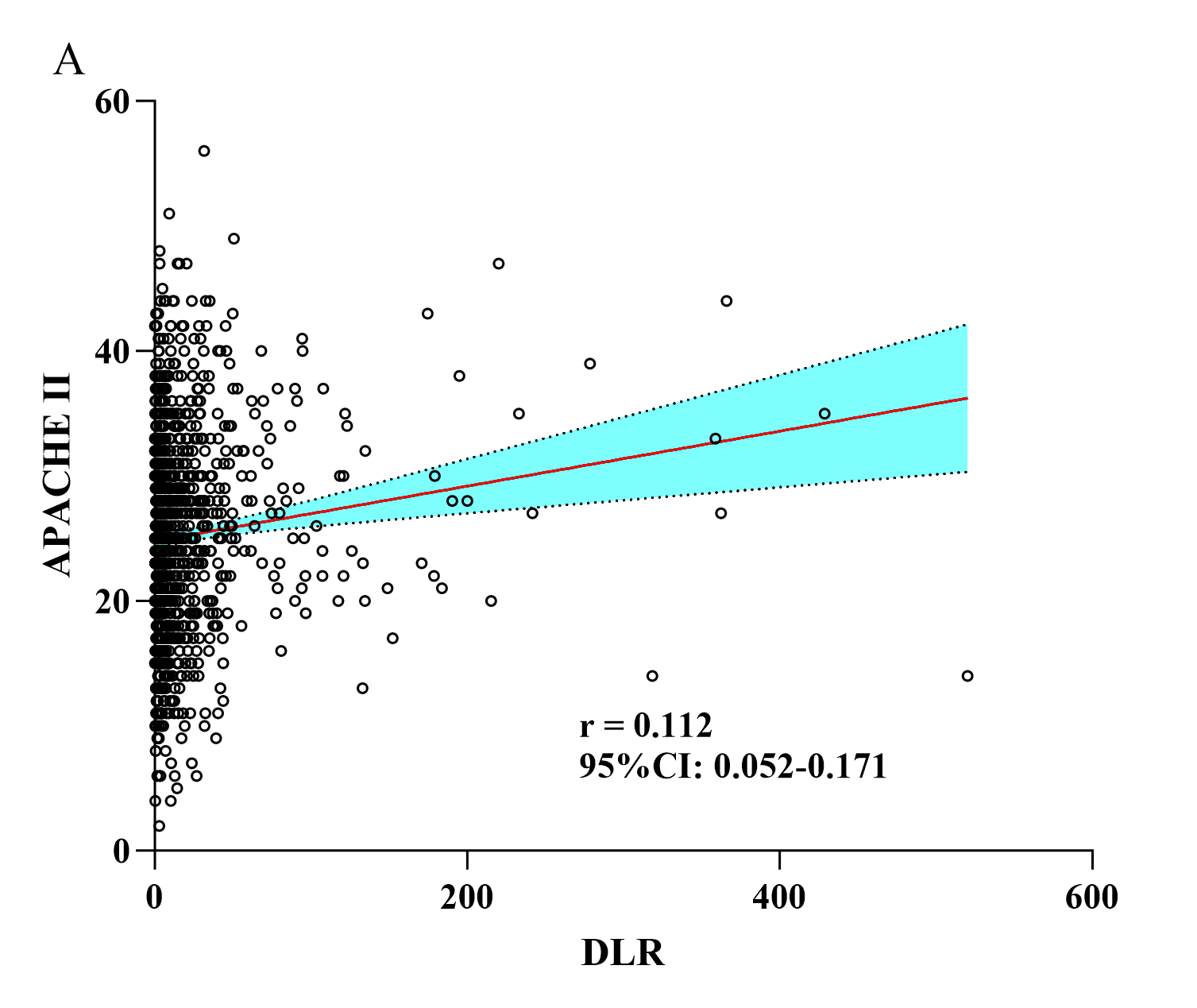

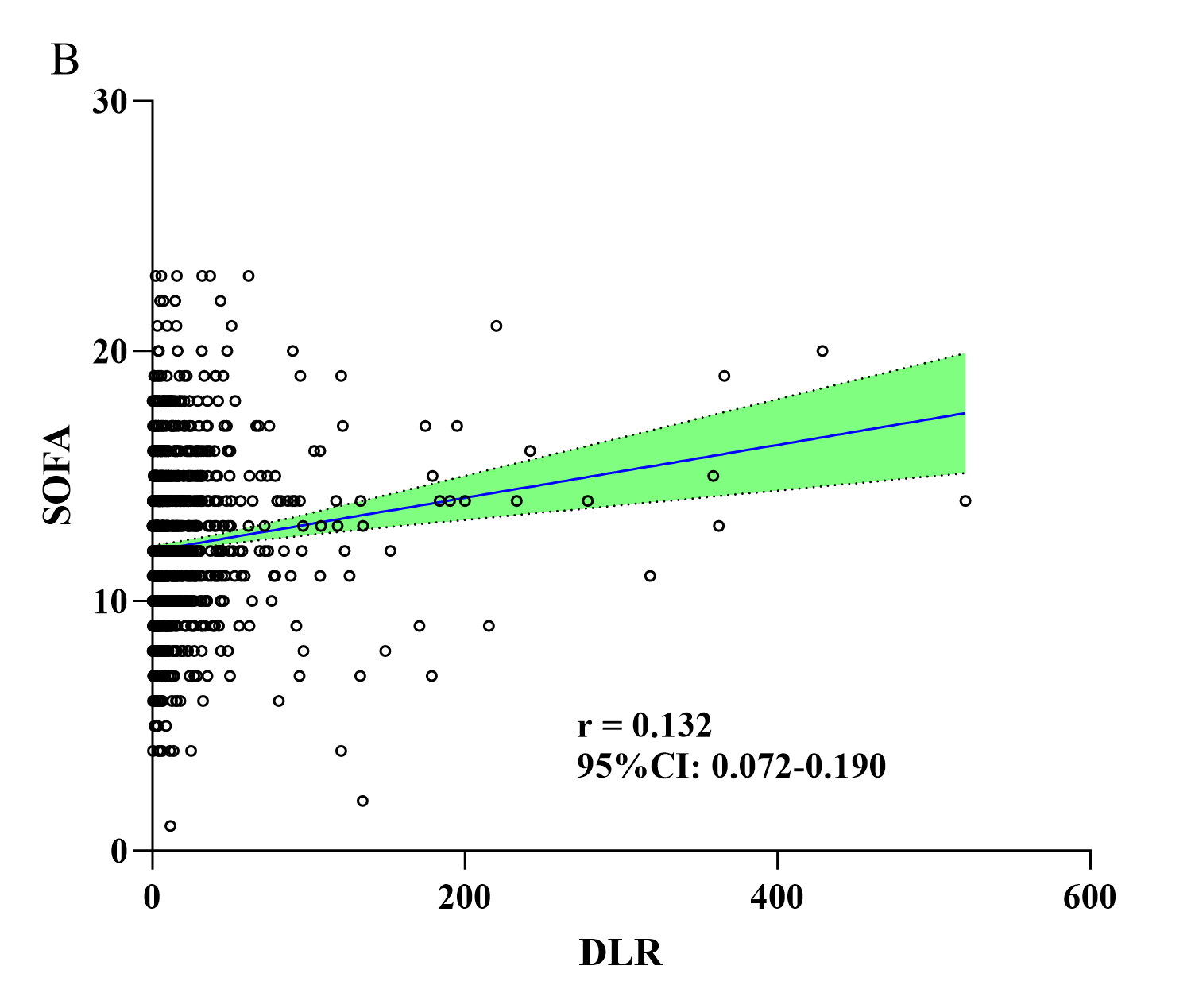


Figure S1. Association between DLR and APACHE II score (A) and SOFA score (B) using Spearman’s analysis. Abbreviations: DLR, D-dimer to lymphocyte ratio; APACHE II, acute physiology and chronic health evaluation II; SOFA, sequential organ failure assessment.

A


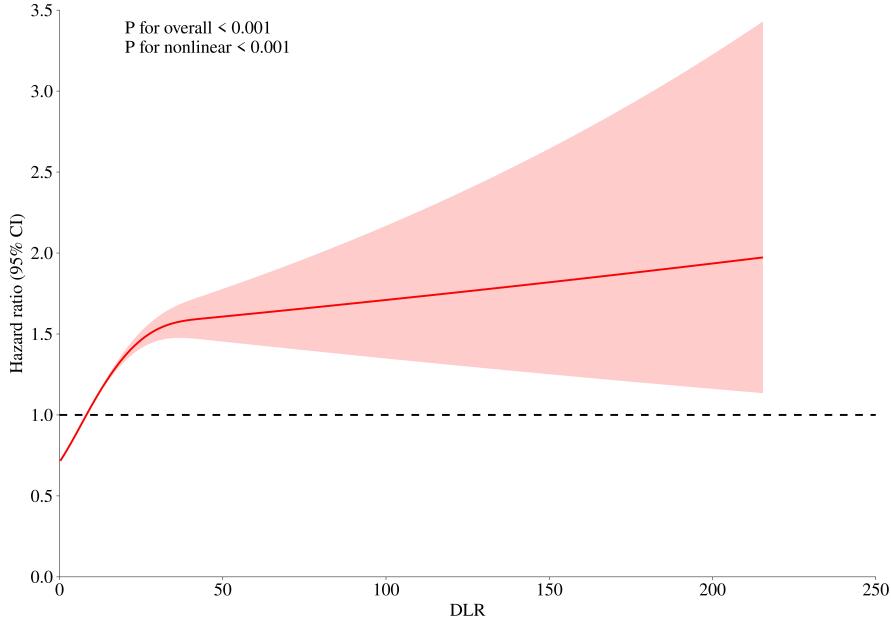


B


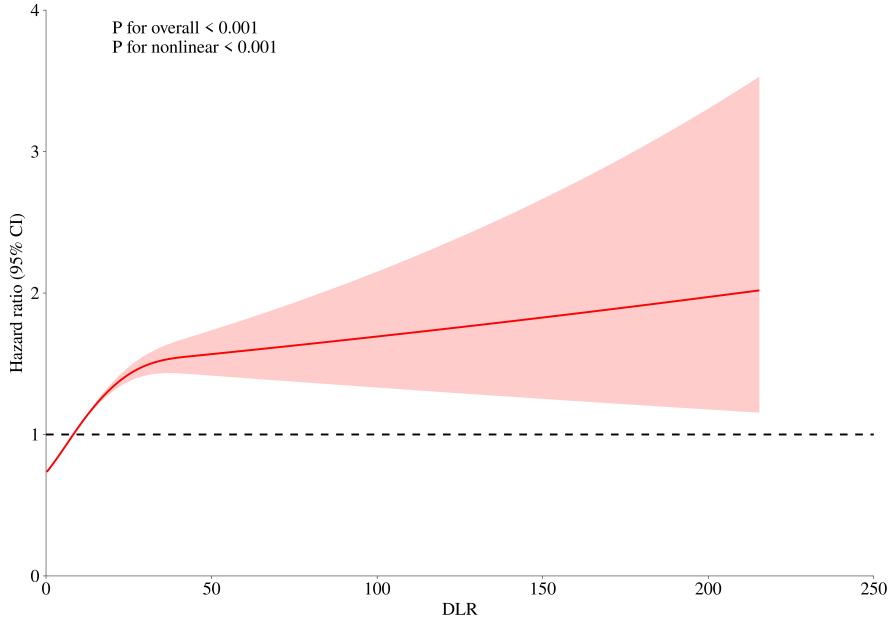


Figure S2. Restricted cubic spline regression analysis of DLR with in hospital all-cause mortality. Heavy central lines represent the estimated adjusted hazard ratios, with shaded ribbons denoting 95% confidence intervals. The horizontal dotted lines represent the hazard ratio of 1.0. A. Restricted cubic spline for hospital mortality. B. Restricted cubic spline for ICU mortality. Abbreviations: DLR, D-dimer to lymphocyte ratio; ICU, Intensive Care Unit.
